# Simulated microgravity increases polyploid giant cancer cells and nuclear localization of YAP

**DOI:** 10.1038/s41598-019-47116-5

**Published:** 2019-07-23

**Authors:** Raj Pranap Arun, Divya Sivanesan, Bamadeb Patra, Sudha Varadaraj, Rama Shanker Verma

**Affiliations:** Stem Cell and Molecular Biology Laboratory, Bhupat and Jyoti Mehta School of Biosciences, Department of Biotechnology, Indian Institute of Technology Madras, Chennai, 600036 TN, India

**Keywords:** Prognostic markers, Macroautophagy, Cancer stem cells

## Abstract

Physical cues are vital in determining cellular fate in cancer. *In vitro* 3D culture do not replicate forces present *in vivo*. These forces including tumor interstitial fluid pressure and matrix stiffness behave as switches in differentiation and metastasis, which are intricate features of cancer stem cells (CSCs). Gravity determines the effect of these physical factors on cell fate and functions as evident from microgravity experiments on space and ground simulations. Here, we described the role of simulation of microgravity (SMG) using rotary cell culture system (RCCS) in increasing stemness in human colorectal cancer cell HCT116. We observed distinct features of cancer stem cells including CD133/CD44 dual positive cells and migration in SMG which was not altered by autophagy induction or inhibition. 3D and SMG increased autophagy, but the flux was staggered under SMG. Increased unique giant cancer cells housing complete nuclear localization of YAP were observed in SMG. This study highlights the role of microgravity in regulating stemness in CSC and importance of physical factors in determining the same.

## Introduction

Therapeutic resistance in cancer is a major hurdle to overcome relapse in patients. Cancer stem cells present in the tumor niche play a vital role in cancer cell self-renewal and chemo/radio-resistance through differentiation of these cells causing invasion and migration leading to secondary tumors. Maintenance and development of stemness properties in cancer cells rely on various intrinsic and extrinsic factors defined by tumor microenvironment. Intrinsic factors include autophagy, cytoskeletal remodeling, metabolic^1,2^ and epigenetic reprogramming etc., Extrinsic factors include matrix stiffness, cell types, and secreted factors, and mechanical signals like^3,4^ TIF (tumor interstitial fluid) pressure which are established *in vivo*^5,6^.

Among extrinsic factors gravity plays a major role in mechanistic signaling, mainly in the form of load bearing. Change in gravity (microgravity/hypergravity) has been shown to affect different cell functions and fate. The microgravity environment induces cell clumping and the reduced force exertion on cells allows for large visible spheroids to form in space. This property of microgravity is exploited in ground experiments to form three dimensional multicellular spheroids. Pioneering studies on mip101 cells, human colorectal cancer cell lines, showed increased cell contact forming large spheroids, structural formations and expression of carcinoembryonic antigen^7^. Variety of cancer cells have been shown to die under microgravity in space and ground experiments^7–11^. The dysfunctional cytoskeleton under SMG is mainly attributed to cancer cell death^11,12^. We observed massive spheroids of Colorectal cancer cells (CRC) which died under Simulated microgravity mediated through G1 cell cycle arrest. But, under the removal of gravitational unloading and exposure to normal conditions showed differentiated phenotype over time^12^. Breast, thyroid and prostate cancer cells have also been shown to differentiate and form unique morphological features when subjected to simulated microgravity^13–15^. Stress signaling and autophagy were found elevated under SMG in various cell types^16,17^. Autophagy has been reported to play crucial role in regulating stemness in stem cells,^2,18^ thus suggesting a transduction between extrinsic physical stimuli and intrinsic changes. Notably autophagy is essential for muscle atrophy and bone loss, hallmark effects of microgravity^19,20^. In this perspective, we questioned the relevance of autophagy regulation under microgravity to understand its effects and cross-relation to physical cues on stemness induction and functional characterization. For better understanding of role of microgravity and segregating effect of microgravity from 3D environment, we cultured cells in RCCS condition (simulated microgravity (SMG)) as well as we grew cells in ultralow attachment dishes to form spheroids (termed as 3D in figures and text). Hippo pathway is involved in regulating apoptosis, stem cell renewal and cell proliferation^21^. The hippo pathway transduces mechanical cues outside the cell to functional outcome through YAP localization. The hippo signaling and autophagy cross-relate through mTORC1-YAP regulating stemness through downstream Yamanaka factors^22,23^. Colorectal cancer is among leading cause of cancer related mortalities^24^, the lining of endothelial cells rich with vascularization makes CRC a viable platform to study environmental factors influencing cancer progression through CSCs and role of gravity in inducing the same. Hence, we studied the CRC stemness under the physical environment of microgravity and its developmental origins through induction of YAP expression and localization.

Our observations based on stemness markers and YAP expression suggest an increase in the presence of cancer stem cells, under SMG. Our results also evaluate the RCCS culture for ground simulation of microgravity as a suitable tool for CSC generation from colorectal cancer cells and studying the biology of cancer stemness.

## Results

### Autophagy flux is staggered under SMG but complete in 3D

**Figure 1:**
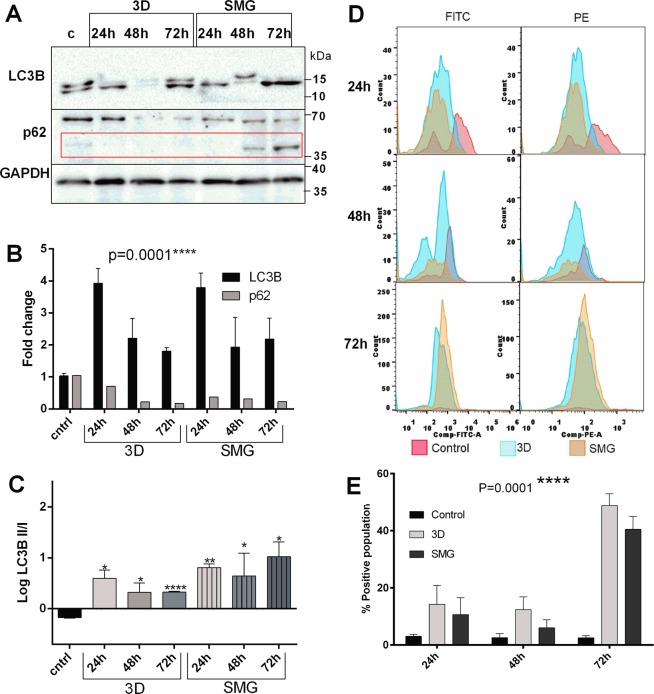
Autophagic Flux is staggered under Simulated Microgravity. Autophagy is significantly increased under 3D and SMG. Western blot images of autophagy markers LC3B, p62 in 3D and SMG at time points 24 h, 48 h and 72 h compared to control (**A**). The red box marks 37 kDa band for native form of p62. The corresponding fold change of expression normalized against GAPDH through densitometry (**B**) represented as mean ± S.D. The LC3B upregulation, P62 downregulation represents increase of autophagy process. The black bars represent relative LC3B expression and lighter bars represent p62 expression. The logarithmic ratio between LC3B-II/LC3B-I represented in graph (**C**). The black bar represent control, the plain light shaded bars represent 3D at 24, 48 and 72 h respectively and the light shaded bars with vertical lines represent SMG at 24,48 and 72 h. The experiment was repeated thrice and the mean ± S.D. of the log ratio was represented. The statistical significance was calculated using unpaired t test with Welch’s correction. Histogram of red and green fluorescence intensity of cells stained with Acridine orange at different time of experimentation (**D**), pink colour represents control cells intensity, blue colour represents cells subjected to 3D and orange colour represent cells subjected to SMG intensity. The representative graph (**E**). The black bars represent control, light bars represent cells subjected to 3D and dark bars represent cells subjected to SMG. The gating of autophagosome positive cell percentage from Acridine orange stained cells was represented in Supplementary Fig. S1. *P < 0.05, **P < 0.005, ***P < 0.001, statistical analysis done using Two-way anova with 95% confidence interval.

Autophagy maintains cellular homeostasis especially in CSCs^2^. Growing cells under hypoxic conditions induce autophagy^25^. Hence, we evaluated autophagy under simulated microgravity and compared against control and 3D using immunoblotting and flow cytometry. Progression of autophagy is marked by (Microtubule associated proteins 1 A/1B light chain 3B) LC3B-I coversion into LC3B-II through conjugation of phosphatidylethanol upregulation and transition of LC3BI to LC3BII^26^. Maturation and completion of autophagy is indicated by degradation of covalently modified sequestrome1 (P62) at around 60 kDa and its unmodified isoforms at 47 and 38 kDa^27^^.^ Western blotting revealed an increased LC3B expression (Fig. 1A, B) and LC3B I to LC3B II transition (Fig. 1C) post 24 h of 3D and SMG, accompanied with P62 degradation. With prolonged duration of 48 and 72 h, variations in total LC3B expression was observed at 48 h and 72 h compared to 24 h without affecting its conversion. P62 expression was reduced throughout the course of the experiment in both 3D and SMG, albeit SMG showed an increase in native form of P62 (37 kDa band) (Fig. 1A) over time (Fig. 1A, B).

**Figure 2:**
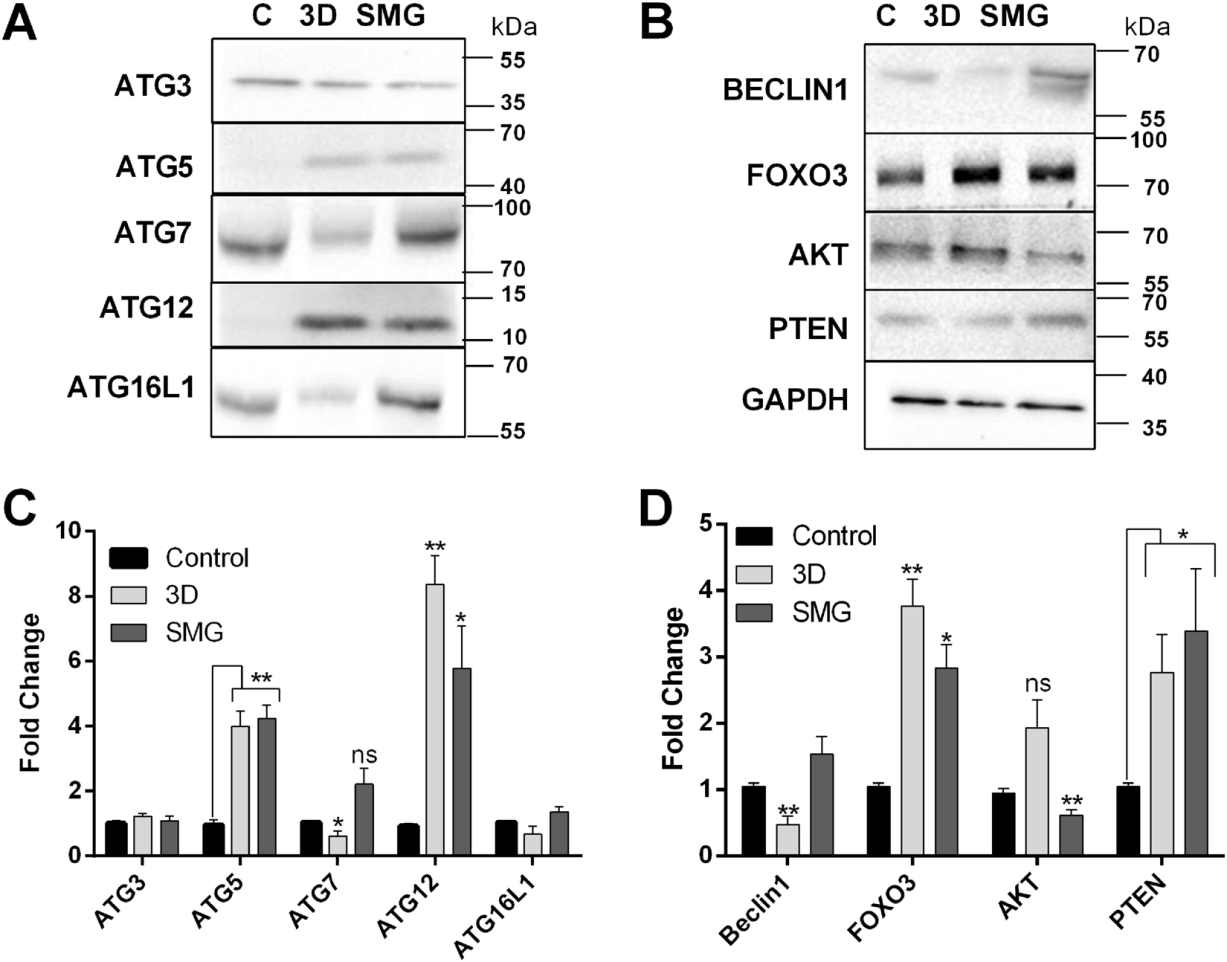
Autophagy core complex proteins are increased under SMG. Western blot images for Autophagic core complex proteins (Autopahgy-Related proteins) ATG3, ATG5, ATG7, ATG12, ATG16L1 at 48 h post 3D or SMG (**A**) and its corresponding graph (**B**) representing mean ± S.D of fold change. Western blot images for autophagy regulator Beclin1 and upstream regulators of autophagy (Forkhead box O3) FOXO3, (protein kinase **B**) AKT, (phosphatase and tensin homolog) PTEN and housekeeping protein GAPDH at 48 hours post 3D or SMG (**C**). the corresponding graph of expression fold change (**D**). The data represented as mean ± S.D. The expression intensity was measured through densitometry of western blot images fold change was normalized against GAPDH. The experiments were repeated thrice *P < 0.05, **P < 0.005, ***P < 0.001, statistical analysis done using unpaired t-test with Welch’s correction.

FACS analysis of autophagosome formation using acridine orange correlated with LC3B expression in both 3D and SMG. The positive population increased with time and at 72 h there was around 40% dual stained cells (Fig. 1D, E). The results reveal a staggered autophagic flux under simulated microgravity. Western blot for ATG (Autophagy related) proteins involved in autophagosome formation and maturation at 48 h further supported this under SMG and 3D. The autophagosome maturation elements ATG5 and ATG12 were upregulated in both 3D and SMG whereas proteins involved in autophagosome formation ATG7, ATG16L1 and Beclin1 were clearly downregulated under 3D, but were mildly elevated in SMG. The upstream signal for autophagosome formation was hindered in 3D via AKT (protein kinase B) upregulation, which was absent in SMG. Upstream elements of autophagy activation including FOXO3 (forkhead box O3), PTEN (phosphatase and tensin homolog) were upregulated in both 3D and SMG, signaling for increased autophagy (Fig. 2).

### Unique CD133^+^/CD44^+^ population are seen in SMG

**Figure 3:**
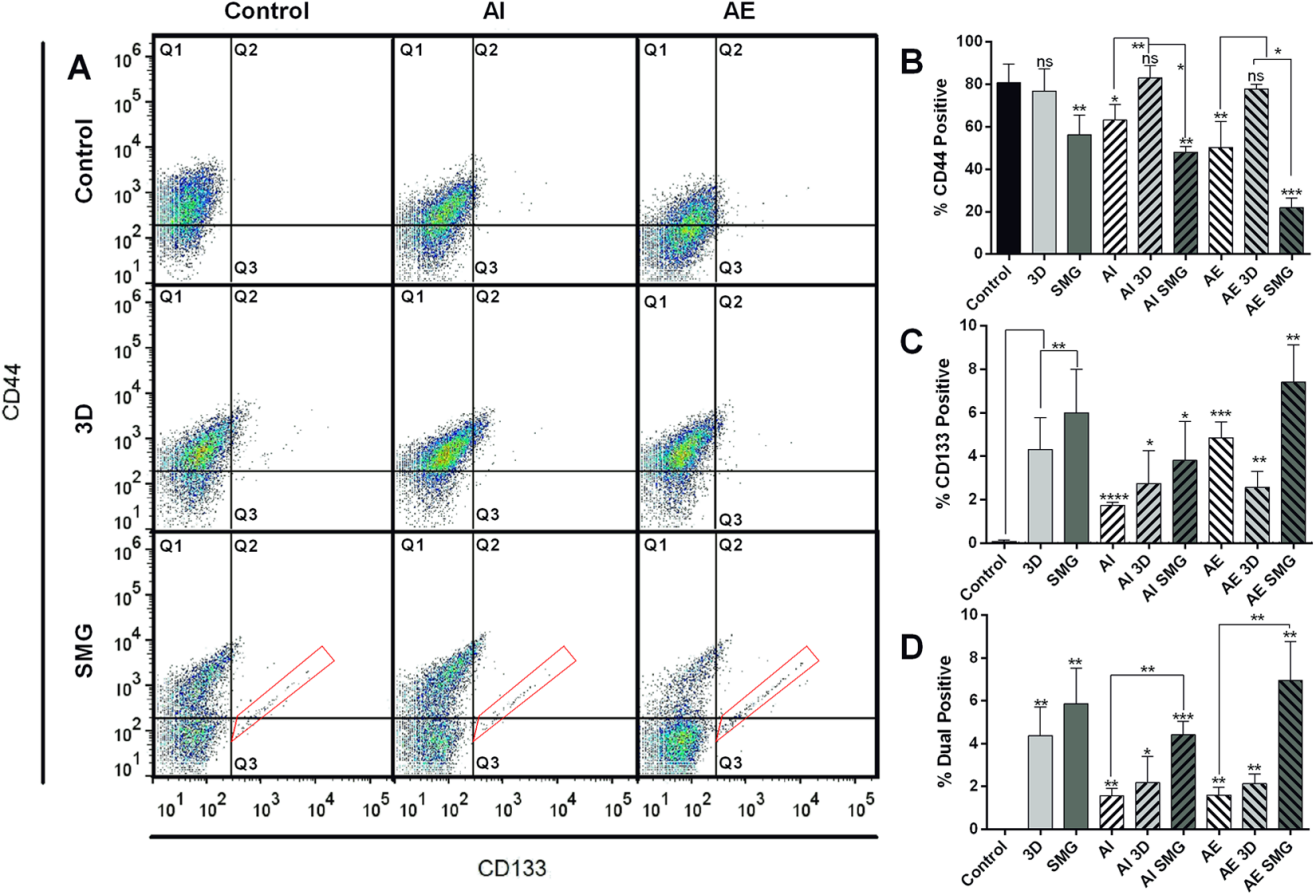
CD133/CD44 expressing cells are more in 3D and SMG. Dot plot for the surface expression of cancer stemness markers CD44 and CD133 analysed with FACS. CD44 stained with FITC, along Y axis and CD133 stained with PE along X axis (**A**). The experimental panel are grouped as rows (no experimental platform 1^st^ row, 3D 2^nd^ row and SMG 3^rd^ row) and treatment groups AI and AE in columns (no treatment 1^st^ column, Bafilomycin treatment (AI) 2^nd^ column and Rapamycin treatment (AE) 3^rd^ column) the top left pane represents control (untreated). The red gating boxed represent unique CD133/CD44 high cells in SMG. The graph representing cells positive for CD44 expression (quadrants 1 + 2) (**B**), cells positive for CD133 expression (quadrants 2 + 3) (**C**) and dual positive population counted form quadrant 2 (**D**). The experiment was repeated thrice and the graphs are represented as mean ± S.D. Significance measured using unpaired t test with Welch’s correction of individual groups with control is represented over the bar and the comparison between AI /AE with 3D/SMG is marked separately. The black bars represent control, light bars represent 3D and dark bars SMG. The bars with (/) lines represent AI and bars with (\) lines represent AE.

We measured stemness in HCT116 cells under SMG and 3D conditions using surface expression analysis of CD133 (prominin 1) and CD44 (hyaluronic acid receptor). To ascertain the role of autophagy, the cells were treated either with rapamycin (to induce autophagy) (AE) or bafilomycin (to inhibit autophagy) (AI). Percentage of CD44^+^ cells was significantly reduced under SMG, AE and AI. Reduction of CD44^+^ cells in AI and AE was rescued by 3D, whereas SMG post AI, AE further reduced the CD44^+^ percentage (Fig. 3A, B). CD133^+^ cells were not seen in control cells, these positive cells increased at 4% and 6% with 3D and SMG respectively, whereas elevation or inhibition of autophagy process resulted in a small increase of ~2%. SMG post AI and AE significantly increased CD133^+^ population to 7% and ~4% respectively (Fig. 3A, C). The reduction in CD44^+^ cells under SMG did not affect dual positive population (Fig. 3D). Unique population of CD133^+^/CD44^+^ cells was observed only in SMG and was not altered much by AI or AE pretreatment gated in red (Fig. 3A).

### Migration correlated with dual positive cells

**Figure 4:**
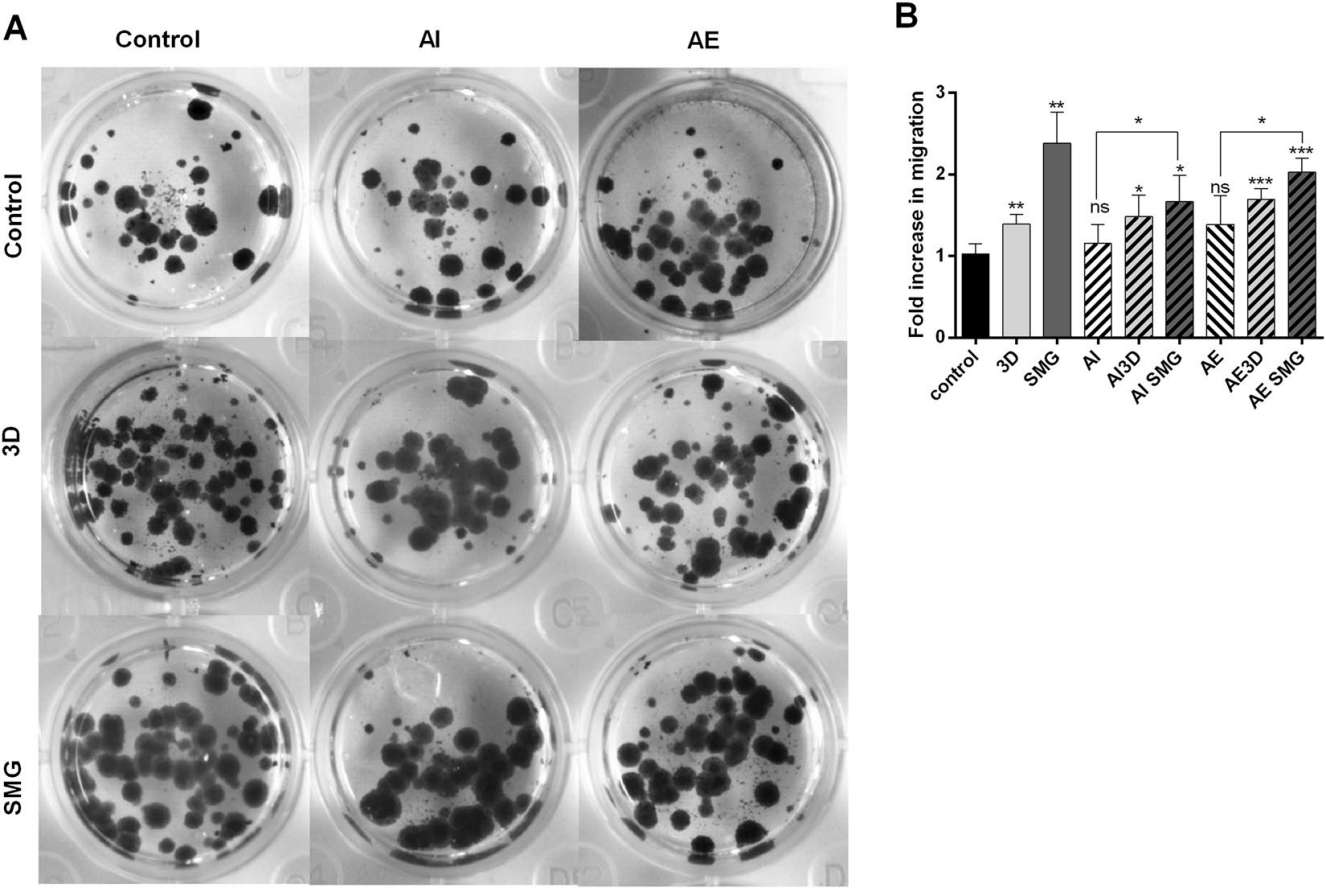
Migration assay. The images of colonies formed from trans-well migration of cells, assayed between 3D and SMG along the rows and treatment for AI and AE along columns against control (top left pane) (**A**). The bar graph representing fold increase in migration compared against control represented as mean fold change ± S.D (**B**). The experiment was repeated thrice, *P < 0.05, **P < 0.005, ***P < 0.001, statistical analysis done using unpaired t test with Welch’s correction. The bars with (/) lines represent AI and bars with (\) lines  represent AE.

To functionally assess dual positive cells, we performed Boyden chamber migration assay. Significant migration was observed in 3D and SMG as compared to control. Modifying the autophagy process did not affect migration, AE showed a small increase in migration ~0.5 fold. Significant increase in migration was observed only in SMG post AI/AE, but not in 3D (Fig. 4A, B). The observed increase in migration directly correlated with CD133^+^/CD44^+^ cells.

### Microgravity induces YAP nuclear localization specifically

**Figure 5:**
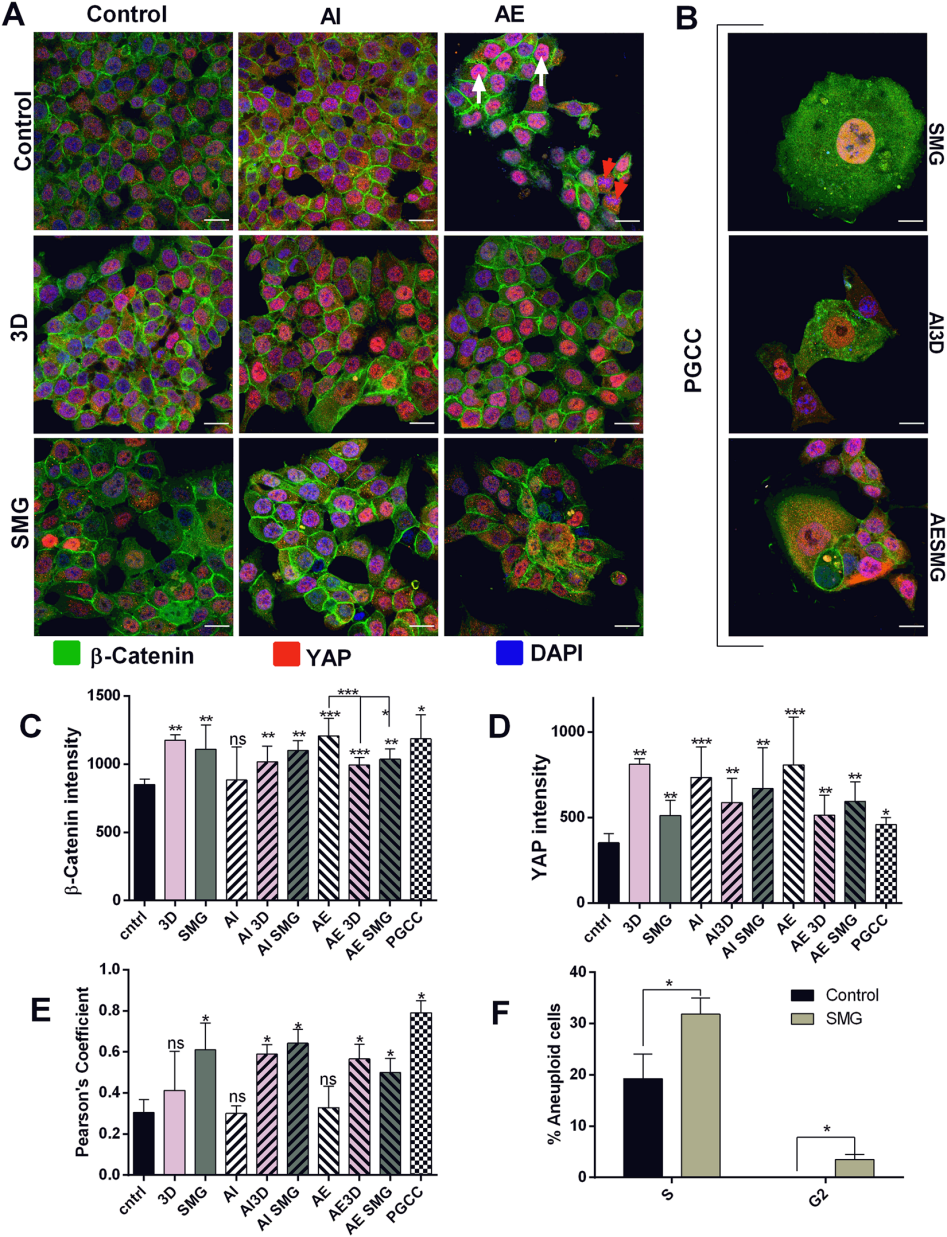
SMG causes YAP nuclear localization and PGCC formation: Confocal images of Stemness regulators β-Catenin and Yes Associated Protein (YAP) stained with FITC (green) and PE (red) respectively, nucleus stained with DAPI (blue) (**A**). The scale bars are 20 µm for all the images. The images were represented with experimental groups 3D and SMG in rows and treatment groups AI and AE in columns. Red arrow heads represent cells in AE with no nuclear YAP and White arrow heads represent cells with nuclear YAP. The confocal images of giant cells observed in SMG cells (**B**) regardless of treatment, with scale bar 20 µm. Bar chart representing average fluorescence intensity for β-Catenin (**C**), and YAP (**D**). The intensity measured between individual cells and the average intensity ± S.D represented. Nuclear localization of YAP measured as terms of Pearson’s coefficient (**E**) represented as mean ± S.D. The bar graphs are represented with control black bars, 3D with light bars and SMG with darker bars. The Bafilomycin treatment AI are represented with (/) lines and Rapamycin treatment (AE) are represented with (\) lines in bars. The checkered bars represent PGCC. Bar graph representing percentage distribution of aneuploid cells among control and SMG with different stages of aneuploid replication (**F**). The light colour depicts SMG and black bar represents control. The experiment was repeated twice and data represented as mean + S.D. statistical analysis performed using unpaired t test with Welch’s correction. *P < 0.05, **P < 0.005, ***P < 0.001, statistical analysis done using Mann Whitney t test for expression intensity (**C**,**D**) and Pearson’s coefficient (**E**).

Yes Associated Protein (YAP) and β-Catenin are known regulators of stemness in cancer cells^28^. β-Catenin signaling is governed by Wnt activation^29^ and Yap signaling by external stimulus such as matrix stiffness^30^. Expression and localization of YAP and β -Catenin in 3D and SMG subjected cells will enlighten the regulatory mechanism for stemness under simulated microgravity. We performed confocal microscopy for YAP stained with PE (red) and β -Catenin stained with FITC (green), co-stained with DAPI (blue) for nuclear localization. β -Catenin was localized to cell membrane and strong in cell contact region and was not altered under any condition as compared to control. Expression of β -Catenin was increased slightly but significantly in all cases except AI, which was also rescued under 3D and SMG (Fig. 5A, C). YAP was majorly localized in the nucleus, but this varied between the experimented conditions. Expression was increased in 3D, AI and AE. The increase of YAP expression in AI and AE was bought down in 3D and in SMG (Fig. 5A, D). Simulated microgravity had a slight increase in expression. Nuclear localization of YAP measured between red and blue by Pearson’s coefficient showed the functional relevance of the expressed YAP. SMG significantly localizes YAP in the nucleus under untreated as well as in AI and AE. 3D also increases nuclear localization, but not as significantly in AI and AE pretreatment. 3D and AE had significant increase in YAP but Pearson’s coefficient was not much altered from control (Fig. 5D, E). Cells with nuclear YAP were visible in AE (white arrows), which was accompanied by cells that completely lack nuclear YAP (red arrows) (Fig. 5A). This contrast in localization resulted in lower Pearson’s coefficient in AE. Unique large cells were observed in SMG, this was not hindered by AI/AE pretreatment. These large cells had abnormally large nuclei and showed similar YAP expression compared to other cells among SMG, but strong YAP nuclear localization (Fig. 5B, E). Previous studies have shown these giant cells to be polyploid giant cancer cells, intricate to cancer stemness^31^. Cell cycle analysis was performed between PI stained cells of control and SMG to analyze ploidy. SMG showed a marked increase of aneuploid S phase and G2 phase cells compared to control, which also housed these aneuploid cells. Aneuploid G2 was observed purely under SMG (Fig. 5F).

### YAP expression and localization triggers Yamanaka factors

**Figure 6:**
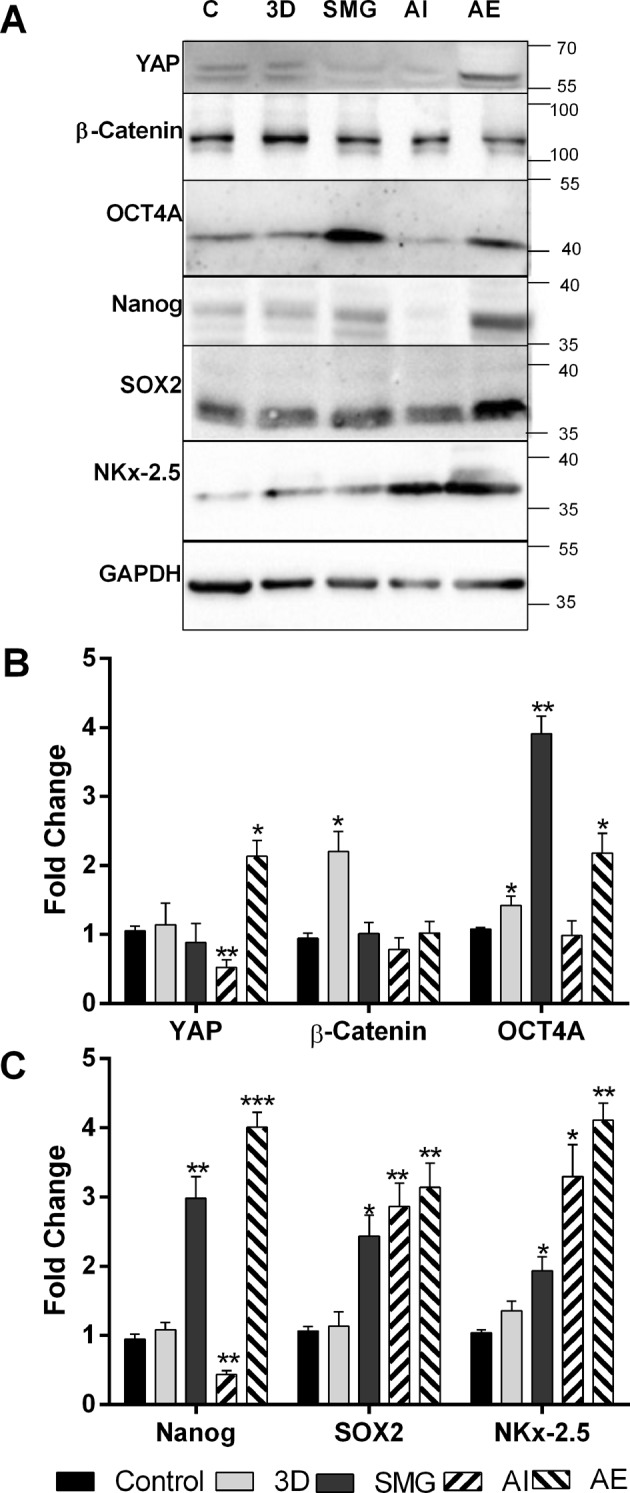
Nuclear YAP causes Yamanaka factor expression. Images for western blot for β-Catenin, (yes associated protein) YAP and Yamanaka factors (Octamer-binding transcription factor 4) OCT4A, (SRY-Box 2) SOX2, (Homeobox protein) Nanog and (NK2 Homeobox 5) NKx-2.5 (**A**) among control, 3D, SMG, Autophagy inhibition and Autophagy elevation. The protein expression represented as fold change normalized against GAPDH, compared against control (**B**) for proteins YAP, β-Catenin and OCT4A and bar graph of protein expression of SOX2, Nanog and NKX2.5 (**C**). The experiments were repeated thrice and the data represented as mean + S.D. *P < 0.05, **P < 0.005, ***P < 0.001, statistical analysis done using unpaired t test with Welch’s correction. The black bars represent control, light bars represent 3D and dark grey bars SMG. The bars with (/) lines represent AI and bars with (\) lines represent AE.

For better understanding of the effect of stemness regulators downstream, we investigated the expression of Yamanaka factors (OCT4A (octamer binding transcription factor 4A), SOX2 (sex determining region box 2), Nanog (homeobox protein NANOG), and NKx-2.5(NK2 homeobox 5)) as well as β-Catenin and YAP by immunoblotting to confirm the expression observed in confocal images. β-Catenin expression was not altered except in 3D, with 2-fold increase. YAP expression was not altered by 3D, SMG and AI but induced by AE. These results were corroborating with immunocytochemistry observations. YAP upregulation in AE resulted in significant elevation of all the 4 Yamanaka factors studied. Inhibition of Autophagy in the other hand significantly reduced OCT4A and Nanog, while SOX2 and NKx2.5 were upregulated significantly. 3D induced no change in Yamanaka factors. SMG upregulated all four factors correlating with the nuclear localized YAP (Fig. 6A–C).

## Discussion

Baseline autophagy is involved in the homeostasis of cell growth and fate^2,18^. Cancer stem cells from various origin have been shown to have increased autophagy, leading to chemoresistance and increased self-renewal. Inhibition of the process or knockdown of proteins involved reverses the same. The increase in LC3B II/I ratio and decreased p62 (60 kDa band) under 3D and SMG suggests that both the physical conditions promote autophagosome maturation and completion of autophagic flux. However, this trend was accompanied with progressive accumulation of unmodified p62 (37 kDa band) with time suggests staggered autophagic flux under SMG, with decline in covalent modifications of p62, an essential event for the targeting of p62 towards ubiquitinylated cargo^32^. This finding differentiates the autophagy changes between 3D and SMG.

The variations in the expression levels of LC3B may be accounted to dynamic changes in autophagy responses in cells subject to stress^33^. Heterogeneous stress conditions of 3D/SMG induced spheroids owing to variations in the amount of physical/nutrient stress at different regions of the spheroids. In this context, the changes in LC3B expression at 48 hrs might account for the cumulative dynamic changes of LC3B expression changes in different cells among the orientation of spheroids, which is not uniform in all experimental duplicates, although maintaining an elevated maturation was uniform among all experimental duplicates indicated by increased LC3II/I ratio.

Studies on autophagy under microgravity dates back to 1981 when Rokhlenko and Mul’diiarov described the increase in autophagosome in myocardial cells subjected to microgravity^34^. But lysosomal content was not significantly altered under microgravity^35^ leading to discredit autophagy role under the reduced gravitational force. However, the levels of autophagic flux as a whole including the autophagosome formation, maturation and clearance has not been clearly established under microgravity. In this perspective, autophagic events requires cytoskeletal integrity^36^. This provides a scenario for the direct effect of cytoskeletal changes associated with SMG in influencing the observed staggered autophagy indicating the differential effect of extrinsic physical features in influencing autophagy progression.

A variety of stem cells have been known to be impeded with changes in the autophagy process, including increase in differentiation of stem cells from mesenchymal, hematopoietic, dermal and epidermal origin with decline in autophagy^37,38^. The main control element of autophagy in stemness maintenance is attributed to inhibition of senescence^39^. Recent evidences suggest a clear relation of autophagy increase in microgravity to the cellular functional processes. Majority of the observed results are focused on muscle atrophy and osteoclastogenesis owing to understand the mechanism of muscle loss during spaceflight and develop interventions for the same^40^. In addition, increase in autophagic process has been attributed to cellular function like increase in osteoclastogenesis in RAW264.7 cells subjected to RCCS culture^17^. Considering the role of autophagy in differentiation and maintenance of stemness, key features of CSCs, we looked into the effect of SMG and 3D on stemness markers viz CD44 and 133 population. Good positive markers are essential in identifying cells with specific functional phenotype/fate. HCT116, with CD44^+^–CD133^+^ - cells are found to be proliferative compared to CD44^low^ CD133^low^ cells, and are high in *in vivo* xenograft tumor formation^41^. Invasion of CD133 positive cells are attributed to TIMP activation^42^. Cellular stresses including ionizing radiation, oxidative stresses and hypoxia activates p53^43^, which in turn mediates CD133 expression and increases cancer stemness^44^. The observed significant increase in CD44^+^ and CD133^+^ population under SMG compared to 3D suggested the presence and activation of control elements for stemness regulation in response to gravitational cues.

CD44 is involved in the attachment of cell on substratum and cell-cell contact and is a key player involved in invasion and migration^45^. Microgravity conditions have cytoskeletal dysfunction^46^ causing major changes in signaling pathway. CD44 was downregulated in SMG, this reduction in CD44 was not affected by increase in autophagy or inhibition of the same. 3D conditions involved tight packing of cells and did not carry the dysfunctional cytoskeleton as seen in SMG, maintained the CD44 expression and also reversed its expression in AI and AE cells. Role of CD44 in growth and migration depends on interaction with surface ligands^47,48^, which in turn requires CD44 recruitment to cell surface. Changes in cytoskeletal organization reduces this^49^. We observed CD44 expression going down in DLD1 cells during SMG^11^. Cytoskeletal dysfunction might be the cause for reduced CD44 expression under SMG. Interestingly the dual positive population (CD133^+^ /CD44^+^) was not hindered by SMG. This can also be attributed to the increased migration of the cells subjected to SMG. It can be considered that the general expression of the CD44 among all the cells in average goes down but the specific cells expressing stemness properties are specifically increased under SMG. Colorectal cancer initiating cells are high CD133^+^ and this is a prognostic marker for lower survival rate^50^. CD133^+^ correlates with increased colony formation and migration in colorectal cancer cells^51^.

Migration showed a direct correlation to presence of CD133^+^/44^+^ dual positive cells. Also, it is important to note that various factors involved in RCCS culture including hypoxia, 3D environment, and modified extracellular matrix each of which can induce migration. RCCS is a low shear environment^10^, the low shear preconditioning might drive for migration. Hence, significantly high migration under SMG might be due to amalgamation of all these migration inducing factors. Autophagy inhibition did not affect migration; previous experiments have shown reduction of migration in gastric cancer cells with bafilomycin treatment compared to autophagy induction^52^. Inhibition of migration is observed through longer rapamycin treatment, whereas acute treatment did not affect HUVEC migration^53^ Longer treatment of rapamycin inhibits migration through negative feed-back of Pi3K-Raf signaling in breast cancer cells MDA-MB 231^54^. Short time exposure to rapamycin in our experiments did not inhibit migration, but instead increased to a small extent.

Localization, and expression of β-Catenin is not much hindered by modifying autophagy and through 3D culture or SMG. The total protein expression of β-Catenin was found high only in 3D owing to the increased cell contact in 3D. Nuclear localization of β-Catenin was completely absent in the experimented conditions, suggesting a minimal Wnt activation in HCT116 cells.

SMG increases aneuploidy, and the presence of giant cells with multiple nuclei (polyploid giant cancer cells (PGCC)). These large cells had abnormally large nucleus, indicating a possibility of failure in the completion of nuclear division or induction of cellular fusion^55^ or intracytoplasmic daughter cell generation as an adaptive resposnse^56^. PGCC in general are observed with endoreplication and display blastomere like developmental pattern^57^. These large cells grew mostly isolated compared to other cells which grew in groups and were very rarely observed in normal conditions or 3D. Isolated growth may cue for cytoplasmic localization of β-Catenin in these cells, cells which were in contact to other cells had β-Catenin aggregation to boundary. The SMG cells had more aneuploid cells and aneuploid G2 population was observed only in SMG, compared to control. Ovarian cancer PGCC derived tumor were CD133^+^/CD44^+^ and exhibit mesenchymal phenotype and chemoresistance^58^.

Sensing of mechanical cues primarily occurs through hippo-YAP signaling resulting in nuclear translocation of YAP under stiffer matrix and cues for a plethora of signaling towards stem cell differentiation and cancer^59^. Clinically, Hippo pathway activation in colorectal cancer is associated with high histological grade tumors with cancer stem cell signature^60^. Also it is important to note that upstream Hippo pathway components are rarely mutated in most cancer types^61^, rendering the downstream YAP signaling regulation through mechanical cues. YAP plays a major role in developmental signaling and mainly in organ size determination^62^. Mesenchymal cell fate under differentiation media is determined through YAP localization^63^. Our previous observation of adipogenic differentiation of mesenchymal stem cells under simulated microgravity^64^ suggested a softer matrix in the cells cultured under SMG. Shifting such cells to normal culture conditions could provide the signals for nuclear localization of YAP and the environment to enrich the formation of PGCC. State of autophagy did not impact PGCC in 3D and control conditions, suggesting the need for mechanistic cues for formation of these cells. The unique CD133^+^/CD44^+^ population was observed only in SMG which might have also risen from the same mechanistic cues, rather than through changes in the autophagic process.

Elevation of Autophagy markedly increased YAP expression, even though the Pearson’s coefficient was low under AE, ratio between the fluorescence intensity between nucleus and cytoplasm was strongly positive in AE suggesting ratiometrically more YAP in nucleus, similar to that of SMG cells. Observations of decreased YAP activation in autophagy compromised cells^65^ fall in line with our observations which further showed a differential YAP localization with autophagy induction using rapamycin. The nuclear localized YAP resulted in downstream signaling with significant increase in all of the Yamanaka factors (OCT4A, SOX2, Nanog, and NKx-2.5). OCT4A and Nanog were nearly absent in AI.

PGCC and YAP signaling are associated with increased expression of Yamanaka factors^65,66^ which are involved in cancer origin in a variety of tumors and these factors lead to higher grade tumors^66–69^.

Collectively these results suggest that, HCT116 cells react to change in gravitational stress with increased but staggered autophagic flux and increased stemness. These results also emphasize the importance of physical cues in formation of CSC with a PGCC phenotype and nuclear YAP localization.

## Conclusion

Microgravity conditions play a vital role in stemness regulators and in upregulation of markers like CD133/CD44, YAP nuclear localization and increase the number of PGCC in culture. The cells cultured under SMG have the unique niche conditions for all the CSC features including Yamanaka factor upregulation and subtype that functionally express in the form of migration. These features were completely absent in control 3D experiments indicating the role of physical niche in elevating stemness in cancer. Further evidence on the CD133^+^/CD44^+^ sorted cells playing role in PGCC formation and YAP localization suggest novel targets towards CSC. Elucidating the cross-relation between the YAP nuclear localization and CD133^+^/CD44^+^ cells in the form of stiffer and softer matrix studies will improve the understanding in the regulatory mechanisms of CSCs and provide insight in the origin of cancer through Yamanaka factors.

## Materials and Methods

### Cell culture

Human colorectal cancer cell line HCT 116 was procured from National Centre for Cell Science, Pune, India. HCT116 is a metastatic cell line was grown in RPMI 1640 medium (Thermo Scientific, USA) supplemented with 10% Fetal Bovine Serum (Thermo Scientific, USA) and 10U/ml of Penicillin, 10 μg/ml of Streptomycin and 2 mM of L-glutamine (Thermo Scientific, USA). Experiments were performed when cells were under logarithmic growth conditions. For autophagy elevation and inhibition, cells were treated with 100 nM rapamycin (Thermo Scientific, USA) and 50 nM bafilomycin (Sigma Aldrich, India) respectively for two hours. For studying the effects of autophagy on 3D or SMG conditions, cells treated with rapamycin AE or Bafilomycin AI were subjected to SMG or 3D.

### 3D culture and simulation of microgravity

The HCT116 cells were seeded into High Aspect Ratio Vessel (HARV) with a Rotary Cell Culture System RCCS (Synthecon, USA) counting 2 × 10^6^ cells/10 ml of media. HARV was run at 10 RPM for 48 hours for migration, confocal, protein expression and CD marker analysis. The time course for autophagy was performed at different time points of 24 h interval for 72 h using different RCCS -HARV for each time point. The media was replaced every 16–24 hours, and additional media was added periodically to avoid air bubble formation or foaming. The clumps formed were harvested without excessive force using a large mouth Pasteur pipette. The clumps were dissociated using 0.25% Trypsin-EDTA (Thermo Scientific, USA) to form single cells, which were used in FACS or seeded in glass slides for confocal assay.

Cells were grown in non-adherent U bottom wells in culture incubator to achieve 3D culture in normal gravity. These wells were created by coating each well of 96 well plate with 50 µl of 0.5% autoclaved agarose, following protocol^70^. The prepared u bottom wells were UV sterilized for 30 minutes and 3500 cells per well were seeded. The plates were centrifuged to make uniform, single spheroid in all the wells instead of clumps and aggregates. The plates were left untouched overnight for spheroid formation, the migration, confocal, CD marker expression and protein expression analysis experiments were performed 48 h post seeding. The autophagy analysis was performed using different plates for each time point at 24 h interval for 72 h. The spheroids were lysed directly for protein isolation or dissociated using 0.25% Trypsin-EDTA to form single cells for FACS analysis and confocal microscopy.

### Ploidy analysis using Propidium Iodide

The control cells and cells post 48 h SMG were harvested and trypsinized to form single cells. 1 × 10^5^ cells were counted, washed in DPBS (Dulbecco’s phosphate buffer saline) and fixed in ice cold ethanol for 30 minutes in 4 °C. The fixed cells were washed in DPBS and centrifuged at 1000RPM for 5 minutes. The cells were incubated with RNase and PI (10 μg/ml and 50 μg/ml respectively) for 30 minutes at 37 °C followed by population distribution of DNA content analysis in BD FACS Canto flow cytometer (BD, USA). Ploidy was analyzed using FCS express software (FCS, USA).

### Protein isolation

The cells were lysed in (Radio Immuno Precipitation Assay) RIPA buffer containing 0.1% sodium deoxycholate, 1 mM EDTA, 1% TritonX-100, 10 mM Tris–Cl, 0.1% SDS, 0.5 mM EGTA, 140 mM NaCl, pH 8.0 along with 1X Protease and Phosphatase Inhibitor Cocktail (Sigma Aldrich, USA). The lysate was incubated at 4 °C with constant agitation in a rotospin (Tarsons, India) at 20 RPM for 30 minutes, followed by centrifugation at 10,000 × g. The supernatant was quantified using BCA (bicinchoninic acid) protein estimation kit (Thermo Scientific, USA). Equal quantities of protein from all the experimental condition were solubilized in sample solubilizing buffer (SSB) for immunoblotting or the lysate was snap-freezed in liquid nitrogen and stored in −20 °C refrigerator for storage.

### Immunoblotting

SDS PAGE (Sodium dodecyl sulfate poly-acrylamide gel electrophoresis) was performed using protein lysates and electro blotted onto 0.22 μm pore sized Nitrocellulose membrane (NCM) (Pall, USA). The blot was blocked in tris-buffered saline with 3% Bovine Serum Albumin (Sigma Aldrich, USA) and 0.2% Tween-20 (Sigma Aldrich, USA) (TBST), washed and incubated in primary antibodies for LC3B, p62, ATG3, ATG5, ATG7, ATG12, ATG16L1, Beclin1, FOXO3, pan AKT, PTEN, OCT4A, SOX2, Nanog, NKx-2.5 and GAPDH (Cell Signalling Technology, USA) for overnight at 4 °C. Following TBST washes, blots were incubated in secondary antibody conjugated with horse-radish peroxidase raised against primary antibody source (Sigma Aldrich, USA) for 1 hour in room temperature. The final wash was given twice with TBST and once in TBS and the protein was probed through enhanced chemiluminescence, using Westar Supernova (Cynagen, Italy). The developed bands were documented using ChemiDoc + XRS (Biorad, USA) and the expression was analysed using Image lab 5.1 (Biorad, USA). GAPDH was used as endogenous control for quantification.

### Immunocytochemistry

Cells were counted and 20,000 cell per well were seeded onto a well of chambered slide and incubate overnight. The cells were fixed in 2% paraformaldehyde for 1 hour in 4 °C and washed with DPBS. After that cells were permeabilized using 0.3% normal goat serum (NGS) in DPBS with 0.025% Triton X for 30 minutes with shaking. The cells were blocked with 0.3% NGS in DPBS and washed thrice with DPBS carefully, followed by YAP and β-catenin antibody incubation. DPBS washing was given followed by fluorescence conjugated secondary antibody incubation for 1 hour. Nuclear staining was done using DAPI in 1:1000 dilution of 2 µg/ml for 15 minutes. The images were taken using FLOWVIEW F3000 confocal microscope (Olympus, USA). Expression and localization were analyzed using ImageJ software through intensity profile and Pearson’s coefficient.

### Flow cytometry analysis

1 × 10^5^ cells of each sample was incubated for 15 minutes in 1:1000 dilution of 1 µg/ml acridine orange and autophagy was quantified following^71^. For CD133 and CD44 expression analysis, 1 × 10^5^cells were blocked for 1 hour in 0.3% NGS for 1 h followed by incubation in manufacturer specified dilution primary antibody (PE conjugated CD44 (BD, USA) and FITC conjugated CD133 (Miltenyi, USA)) for 1 hour. The cells were washed and the expression was measured in BD FACS CANTOII flow cytometer (BD, USA). Viable cells were gated and the expression of CD44 and CD133 were measured post compensation. The analysis was performed using FLowJo software (Flowjo USA).

### Migration assay

20,000 live cells were counted using trypan blue staining and seeded into the top chamber of the Boyden chamber in serum free media. The bottom chamber was filled with media containing 20% FBS. The cells were allowed to migrate for 48 hours after which the Boyden chambers were removed. The migrated cells were grown in normal growth media for 72 hours and the resultant colonies formed were fixed and stained using crystal violet (0.5% in 25% methanol w/v). The excess stain was washed in 50% ethanol in DPBS and images of each well were obtained. The area of cell growth was compared between control and test conditions using ImageJ software.

### Statistical analysis

Individual experiments were repeated at-least thrice with individual controls for each experiment. The resultant data was analyzed using GraphPad Prism version 6.01 (GraphPad software, La Jolla California USA). Significance was tested using unpaired t-test with Welch’s correction for western blots and FACS expression analysis. Mann-Whitney T test was used for intensity profile and Pearson’s coefficient. Two-way anova with 95% confidence interval was used in time point assay for autophagy. The results were depicted as mean ± S.D.

## Supplementary information

Supplementary figures
