# Supplementary material for: Simulated microgravity increases polyploid giant cancer cells and nuclear localization of YAP
Source: Sci Rep. 2019 Jul 23;9:10684. doi: 10.1038/s41598-019-47116-5 (PMC6650394; doi:10.1038/s41598-019-47116-5)
Supplement: Supplementary file 1 — Supplementary figures [file 41598_2019_47116_MOESM1_ESM.docx]

**Supplementary information**

**Simulated microgravity increases polyploid giant cancer cells and nuclear localization of YAP**

Authors

Raj Pranap Arun, Divya Sivanesan, Bamadeb Patra, Sudha Varadaraj, Rama Shanker Verma*

Stem Cell and Molecular Biology Laboratory, Bhupat and Jyoti Mehta School of Biosciences, Department of Biotechnology, Indian Institute of Technology Madras.

*Corresponding Author

Dr. Rama Shanker Verma

Block 1, Room No.201, Department of Biotechnology

Bhupat and Jyoti Mehta School of Biosciences

Indian Institute of Technology Madras, Chennai, India, 600036

phone: 91-44-22574109,

e-mail: vermars@iitm.ac.in


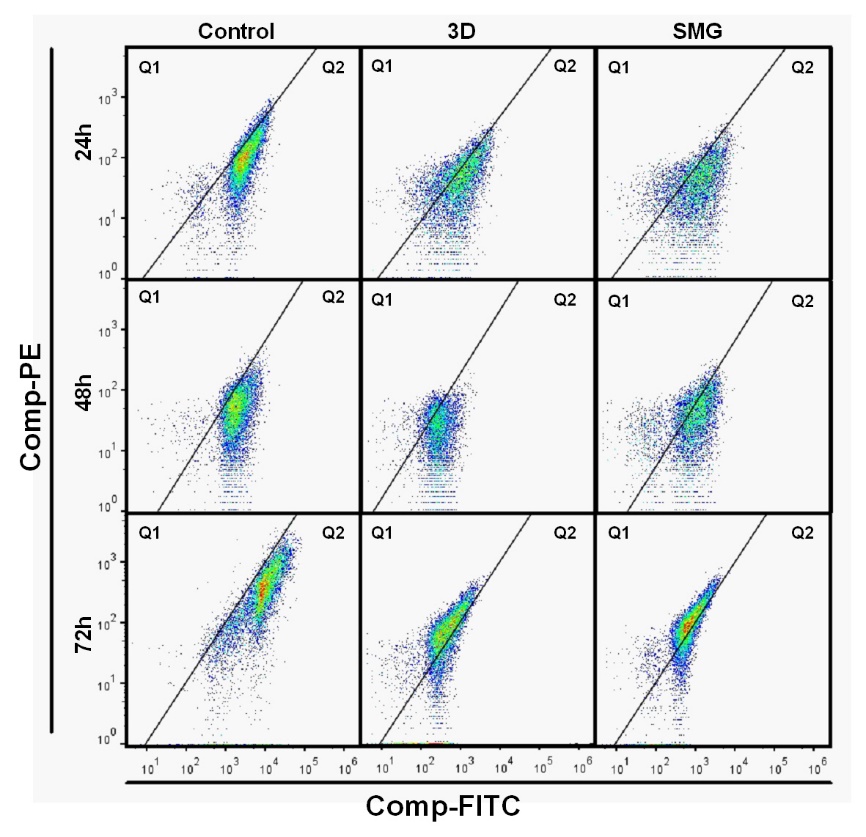


**Supplementary figure1: Gating for Autophagy positive cells**

Dot plot images of Acridine orange stained cells compensated between FITC and PE. The X-axis represents FITC and Y-axis represents PE. The three different time points T1 (24 hours), T2 (48 hours) and T3 (72 hours) represented row wise, and the experimental group control, 3D and SMG represented column wise. The cells gated on the left side of each pane (Q1) is positive for autophagy. The experiment was repeated thrice with individual controls to accommodate for voltage gating.


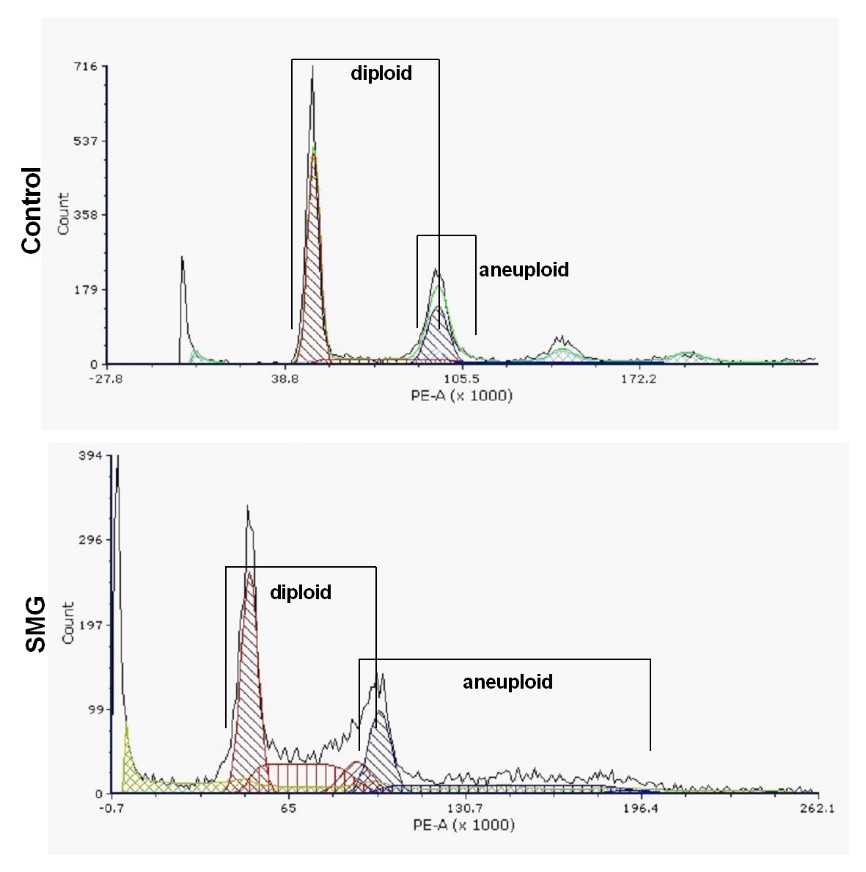


**Supplementary figure 2: Ploidy analysis**

Histograms for ploidy analysis between control and SMG. The aneuploid population is represented by blue shaded lines, euploid population is marked by red shaded lines. Control in the top pane and SMG in the bottom pane.
